# Chapter 4: Protein Interactions and Disease

**DOI:** 10.1371/journal.pcbi.1002819

**Published:** 2012-12-27

**Authors:** Mileidy W. Gonzalez, Maricel G. Kann

**Affiliations:** 1 National Center for Biotechnology Information, National Library of Medicine, National Institutes of Health, Bethesda, Maryland, United States of America; 2 Biological Sciences, University of Maryland, Baltimore County, Baltimore, Maryland, United States of America; Whitehead Institute, United States of America

## Abstract

Proteins do not function in isolation; it is their interactions with one another and also with other molecules (e.g. DNA, RNA) that mediate metabolic and signaling pathways, cellular processes, and organismal systems. Due to their central role in biological function, protein interactions also control the mechanisms leading to healthy and diseased states in organisms. Diseases are often caused by mutations affecting the binding interface or leading to biochemically dysfunctional allosteric changes in proteins. Therefore, protein interaction networks can elucidate the molecular basis of disease, which in turn can inform methods for prevention, diagnosis, and treatment. In this chapter, we will describe the computational approaches to predict and map networks of protein interactions and briefly review the experimental methods to detect protein interactions. We will describe the application of protein interaction networks as a translational approach to the study of human disease and evaluate the challenges faced by these approaches.

**What to Learn in This Chapter**

- Experimental and computational methods to detect protein interactions
- Protein networks and disease
- Studying the genetic and molecular basis of disease
- Using protein interactions to understand disease

This article is part of the “Translational Bioinformatics” collection for *PLOS Computational Biology*.

## 1. Introduction

Early biological experiments revealed proteins as the main agents of biological function. As such, proteins ultimately determine the phenotype of all organisms. Since the advent of molecular biology we have learned that proteins do not function in isolation; instead, it is their interactions with one another and also with other molecules (e.g. DNA, RNA) that mediate metabolic and signaling pathways, cellular processes, and organismal systems.

protein interaction” is generally used to describe the physical contact between proteins and their interacting partners. Proteins associate physically to create macromolecular structures of various complexities and heterogeneities. Proteins interact in pairs to form dimers (e.g. reverse transcriptase), multi-protein complexes (e.g. the proteasome for molecular degradation), or long chains (e.g. actin filaments in muscle fibers). The subunits creating the various complexes can be identical or heterogeneous (e.g. homodimers vs. heterodimers) and the duration of the interaction can be transient (e.g. proteins involved in signal transduction) or permanent (e.g. some ribosomal proteins). However, protein interactions do not always have to be physical [1]. The term “protein interaction” is also used to describe metabolic or genetic correlations, and even co-localizations. Metabolic interactions describe proteins involved in the same pathway (e.g. the Krebs cycle proteins), while genetically identified associations identify co-expressed or co-regulated proteins (e.g. enzymes regulating the glycolytic pathway). As the name implies, protein interactions by co-localization list proteins found in the same cellular compartment.

**Figure 1:**
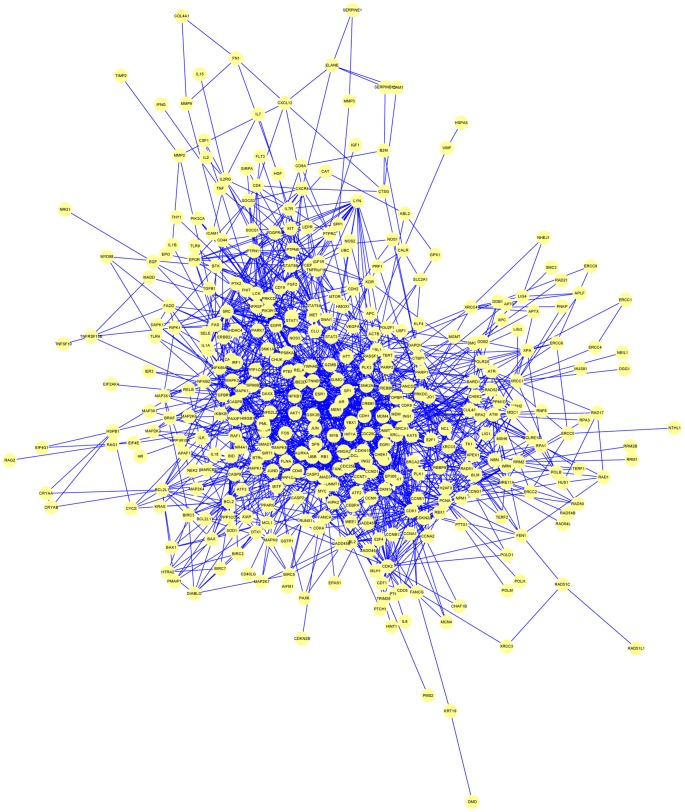
A PPI network of the proteins encoded by radiation-sensitive genes in mouse, rat, and human, reproduced from [**89**]. Yellow nodes represent the proteins and blue lines show the interactions between them. The radiation-related genes were text-mined from PubMed and the protein interaction information was obtained from HPRD.

Whether the association is physical or functional, protein-protein interaction (PPI) data can be used in a larger scale to map networks of interactions [2], [3]. In PPI network graphs, the nodes represent the proteins and the lines connecting them represent the interactions between them (Figure 1). Protein interaction networks are useful resources in the abstraction of basic science knowledge and in the development of biomedical applications. By studying protein interaction networks we can learn about the evolution of individual proteins and about the different systems in which they are involved. Likewise, interaction maps obtained from one species can be used, with some limitations, to predict interaction networks in other species. Protein interaction networks can also suggest functions for previously uncharacterized proteins by uncovering their role in pathways or protein complexes [4]. Due to their central role in biological function, protein interactions also control the mechanisms leading to healthy and diseased states in organisms. Diseases are often caused by mutations affecting the binding interface or leading to biochemically dysfunctional allosteric changes in proteins. Therefore, protein interaction networks can elucidate the molecular basis of disease, which in turn can inform methods for prevention, diagnosis, and treatment [5], [6].

The study of human disease experienced extensive advancements once the biomedical characterization of proteins shifted to studies taking into account a protein's network at different functional levels (i.e. in pair-wise interactions, in complexes, in pathways, and in whole genomes). For instance, consider how our understanding of Huntington's disease (HD) has evolved from the early Mendelian single-gene studies to the latest HD-specific network-based analyses. HD is an autosomal dominant neurodegenerative disease with features recognized by Huntington in 1872 [7], and whose specific patterns of inheritance were documented in 1908 [8]. After almost a century of genetics studies, the culprit gene in HD was identified; in 1993, we learned that HD was caused by the repeat expansion of a CAG trinucleotide in the Huntingtin (*Htt*) gene [9]. This expansion causes aggregation of the mutant *Htt* in insoluble neuronal inclusion bodies, which consequently leads to neuronal degeneration. Yet, even when the key disease-causing protein in HD had been identified, the mechanism for *Htt* aggregation remained unknown. In 2004, Goehler *et al.* [10] mapped all the PPIs that take place in HD and discovered that the interaction between *Htt* and GIT1, a GTPase-activating protein, mediates *Htt* aggregation. Further validation ([11], [12]) confirmed GTI1's potential as a target for therapeutic strategies against HD.

In this chapter, we will describe the main experimental methods to identify protein interactions and the computational approaches to map their networks and to predict new interactions purely *in silico*. We will describe the application of protein interaction networks as a translational approach to the study of human disease and evaluate the challenges faced by these approaches.

## 2. Experimental Identification of PPIs

### 2.1 Biophysical Methods

**Figure 2:**
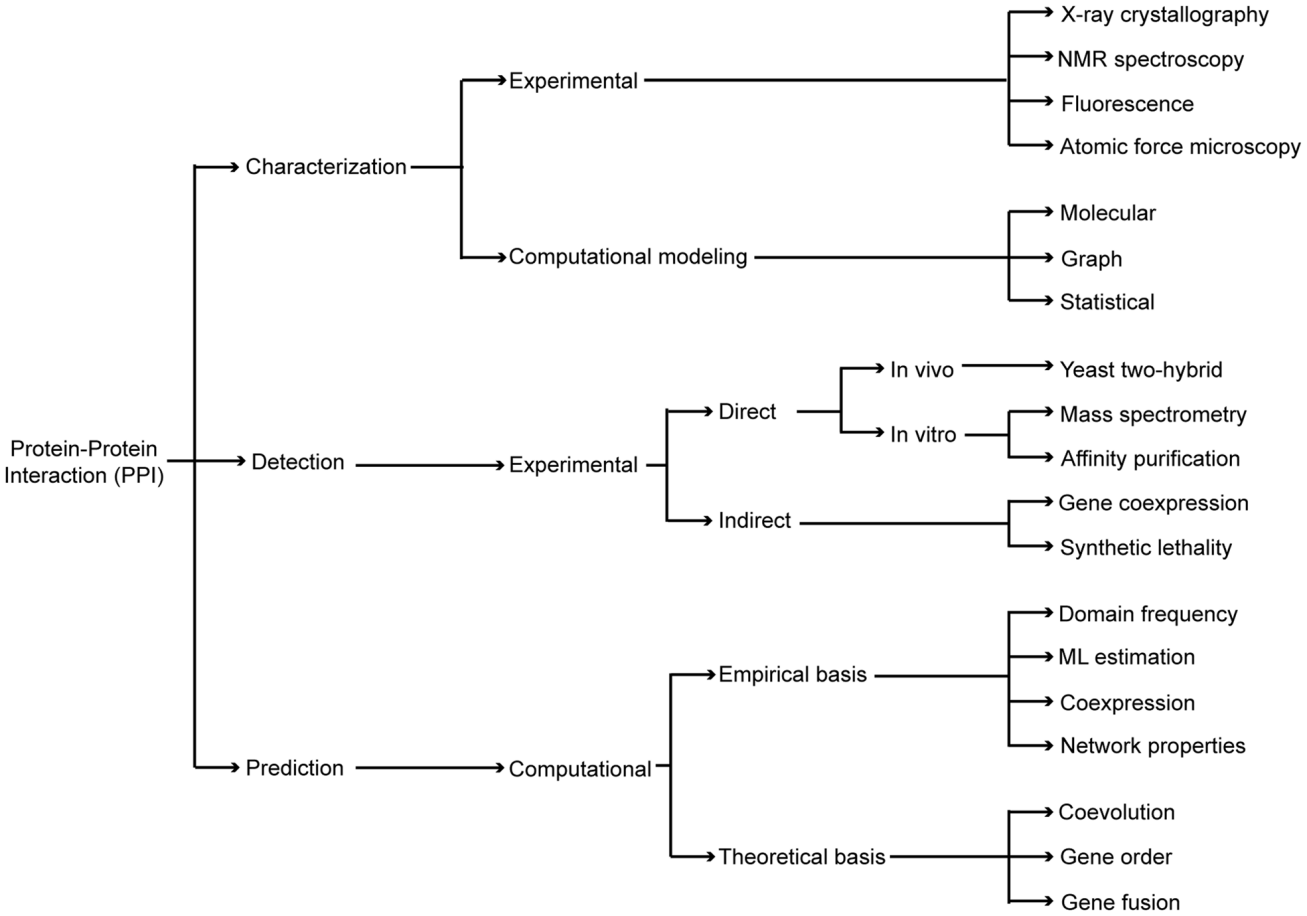
A diagram of the different experimental and computational methods to characterize, detect, and predict PPIs.

Protein interactions are identified through different biochemical, physical, and genetic methods (Figure 2). Historically, the main source of knowledge about protein interactions has come from biophysical methods, particularly from those based on structural information (e.g. *X-ray crystallography*, *NMR spectroscopy*, *fluorescence*, *atomic force microscopy*). Biophysical methods identify interacting partners and also provide detailed information about the biochemical features of the interactions (e.g. binding mechanism, allosteric changes involved). Yet, since they are time- and resource-consuming, biophysical characterizations only permit the study of a few complexes at a time.

### 2.2 High-Throughput Methods

To document protein interactions at a larger scale, automated methods have been developed to detect interactions directly or to deduce them through indirect approaches (Figure 2).

#### 2.2.1 Direct high-throughput methods

*Yeast two-hybrid* (Y2H) is one of the most-commonly used direct high-throughput method. The Y2H system tests the interaction of two given proteins by fusing each of them to a transcription-binding domain. If the proteins interact, the transcription complex is activated, which transcribes a reporter gene whose product can be detected. Since it is an *in vivo* technique, the Y2H system is highly effective at detecting transient interactions and can be readily applied to screen large genome-wide libraries (e.g. to map an organisms' full set of interactions or interactome). But, the Y2H system is limited by its biases toward non-specific interactions. Likewise, Y2H cannot identify complexes (i.e. it only reports binary interactions) or interactions of proteins initiating transcription by themselves. Although protein interactions are usually detected and studied in pair-wise form, in reality they often occur in complexes and as part of larger networks of interaction. *In vitro* direct detection methods (e.g. *mass spectrometry*, *affinity purification*) are better suited to detect macromolecular interactions, yet, they have their own limitations: interactions occurring *in vitro* do not necessarily occur *in vivo* (e.g. when proteins are compartmentalized in different cell locations) and complexes are often difficult to purify, which is a required step in the protocol [13].

#### 2.2.2 Indirect high-throughput methods

Several high-throughput methods deduce protein interactions by looking at characteristics of the genes encoding the putative interacting partners. For instance, *gene co-expression* is based on the assumption that the genes of interacting proteins must be co-expressed to provide the products for protein interaction. Expression profile similarity is calculated as a correlation coefficient between relative expression levels and subsequently compared against a background distribution for random non-interacting proteins. *Synthetic lethality*, on the other hand, introduces mutations on two separate genes, which are viable alone but lethal when combined, as a way to deduce physically interacting proteins [14].

## 3. Computational Predictions of PPIs

As discussed in section 2, experimental approaches provide the means to either empirically characterize protein interactions at a small scale or to detect them at a large scale. Still, experimental detections only generate pair-wise interaction relationships and with incomplete coverage (because of experimental biases toward certain protein types and cellular localizations). Experimental identification methods also exhibit an unacceptably high fraction of false positive interactions and often show low agreement when generated by different techniques [15]–[17]. Experimental biophysical methods can complement the high-throughput detections by providing specific interaction details; but they are expensive, extremely laborious, and can only be implemented for a few complexes at a time.

Computational methods for the prediction of PPIs provide a fast and inexpensive alternative to complement experimental efforts. Computational interaction studies can be used to validate experimental data and to help select potential targets for further experimental screening [18]. More importantly, computational methods give us the ability to study proteins within the context of their interaction networks at different functional levels (i.e. at the complex, pathway, cell, or organismal level), thus, allowing us to convert lists of pair-wise relationships into complete network maps. Since they are based on different principles, computational techniques can also uncover functional relationships and even provide information about interaction details (e.g. domain interactions), which may elude some experimental methods.

### 3.1 Computational Methods for PPI Predictions

Computational interaction prediction methods can be classified into two types: methods predicting protein domain interactions from existing empirical data about protein-protein interactions and methods relying entirely on theoretical information to predict protein-protein or domain-domain interactions (Figure 2).

#### 3.1.1 Empirical predictions

The computational techniques based on experimental data use the *relative frequency of interacting domains* [19], *maximum likelihood estimation of domain interaction probability* [20], [21], *co-expression* [22], or *network properties* [23]–[27] to predict protein and domain interactions. The main disadvantage of empirical computations is that, by relying on an existing protein network to infer new nodes, they propagate the inaccuracies of the experimental methods.

#### 3.1.2 Theoretical predictions

Theoretical techniques to predict PPIs incorporate a variety of biological considerations; they take advantage of the fact that interacting proteins coevolve to preserve their function (e.g. *mirrortree*, *phylogenetic profiling* [28]–[35]), occur in the same organisms (e.g. [36], [37]), conserve gene order (e.g. *gene neighbors method* [38], [39]) or are fused in some organisms (e.g. the *Rosetta Stone method* [40], [41]).

### 3.2 Theoretical Predictions of PPIs Based on Coevolution

Below, we will expand on two methods generating theoretical PPI predictions through coevolutionary signal detection either at the residue or at the full-sequence level.

#### 3.2.1 Coevolution at the residue level

Pairs of residues within the same protein can coevolve because of three-dimensional proximity or shared function [42]. The intramolecular correlations of interacting protein partners can be used to predict intermolecular coevolution. Residue-based coevolution methods measure the set of correlated pair mutations in each protein. A pair of proteins is assumed to interact if they show enrichment of the same correlated mutations [42].

#### 3.2.2 Coevolution at the full-sequence level

**Figure 3:**
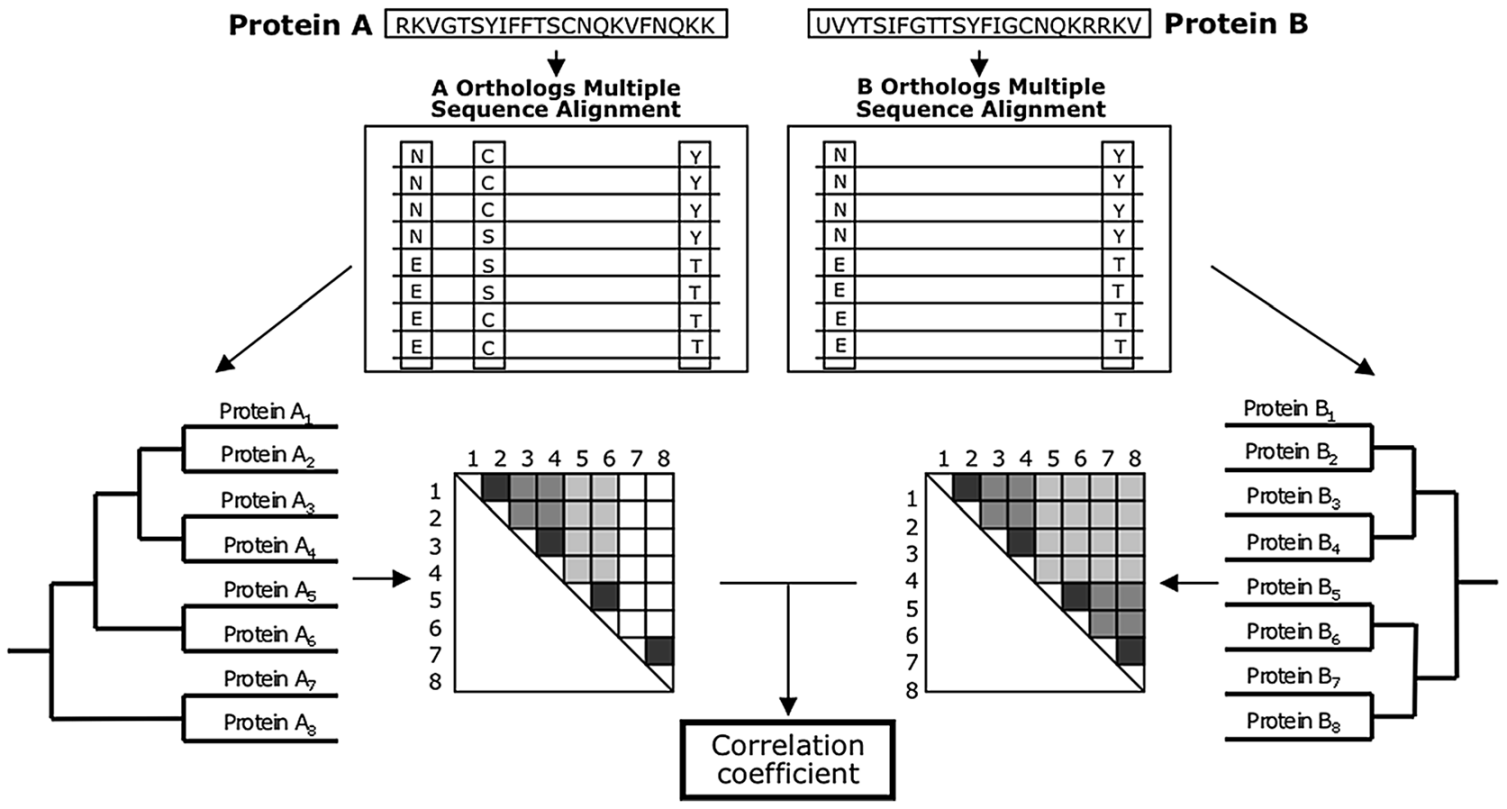
A schema of the mirrortree method for predicting interacting proteins. The orthologs of two proteins (A and B from the same species) are used to construct two multiple sequence alignments (MSAs). Distance matrices, which implicitly represent evolutionary trees, are constructed from the MSAs. Each matrix square represents the tree distance between two orthologs and dark colors represent closeness. The two distance matrices are compared using linear correlation. A high correlation between the distance matrices suggests interaction between proteins A and B.

Methods detecting coevolution at the full-sequence level are based on the idea that changes in one protein are compensated by correlated changes in its interacting partner to preserve the interaction [29], [30], [42]–[45]. Therefore, as interacting proteins coevolve, they tend to have phylogenetic trees with topologies that are more similar than expected by chance [46]. The coevolution of interacting proteins was first qualitatively observed for polypeptide growth factors, neurotransmitters, and immune system proteins with their respective receptors [47]. Several methodologies have been developed to measure coevolution at the full-sequence level, and among them, the mirrortree method is one of the most intuitive and accurate options. As shown in Figure 3, mirrortree measures coevolution for a given pair of proteins by i) identifying the orthologs of both proteins in common species, ii) creating a multiple sequence alignment (MSA) of each protein and its orthologs, iii) from the MSAs, building distance matrices, and iv) calculating the correlation coefficient between the distance matrices. The mirrortree correlation coefficient is used for measuring tree similarity, thereby, allowing the evaluation of whether the proteins in question coevolved [28]–[35].

The mirrortree method has been successfully implemented to confirm experimental interactions in *E. coli* [4], *S. cerevisiae* [48], and *H. sapiens* [49]. But, the degree of similarity between the phylogenetic trees is strongly affected by the sequence divergence driven by the underlying speciation process [4], [50]. Therefore, two proteins may have similar phylogenetic trees due only to common speciation events, but they may not necessarily be interacting partners. By subtracting the signal from speciation events Pazos *et al.* [4] and Sato *et al.* [50] showed improvements for the performance of the mirrortree method. One approach creates a “speciation” vector from the distance matrices derived from the ribosomal 16S sequences (for prokaryotes and 18S for eukaryotes), while the other uses the average distance of all proteins in a pair of organisms. Both methods subtract the speciation vector from the original distance matrix constructed for the given protein pair.

In principle, to characterize protein interactions at a systems level, all protein-protein and domain-domain interactions in a given organism must be catalogued. The mirrortree method is a suitable option to complement experimental detections because it is inexpensive and fast. Moreover, mirrortree only requires the proteins' sequences as input and thus can be used to analyze proteins for which no other information is available. Since mirrortree predictions are based on different principles than any other computational or experimental techniques, they can also uncover functional relationships eluding other methods. Still, the implementation of the mirrortree approach is under several limitations. One limitation of the mirrortree method is the minimum number of orthologs it requires. Selecting orthologs in large families with many paralogs is also a considerable challenge for mirrortree [49]. In addition, coevolution does not necessarily take place uniformly across the sequence; different sites may coevolve at different rates based on functional constraints. Thus, coevolution signals vary when measured across the entire sequence vs. at the domain level [51].

## 4. Protein Networks and Disease

### 4.1 Studying the Genetic Basis of Disease

The majority of our current knowledge about the etiology of various diseases comes from approaches aiming to uncover their genetic basis. In the near future, the ability to generate individual genome data using next generation sequencing methods promises to change the field of translational bioinformatics even more.

Since the inception of Mendelian genetics in the 1900's, great effort has gone into cataloguing the genes associated with individual diseases. A gene can be isolated based on its position in the chromosome by a process known as positional cloning [52]. A few examples of human disease-related genes identified by positional cloning include the genes associated with cystic fibrosis [53], HD [9], and breast cancer susceptibility [54], [55]. Even in simple Mendelian diseases, however, the correlation between the mutations in the patient's genome and the symptoms is not often clear [56]. Several reasons have been suggested for this apparent lack of correlation between genotype and phenotype, including pleiotropy, influence of other genes, and environmental factors.

*Pleiotropy* occurs when a single gene produces multiple phenotypes. Pleiotropy complicates disease elucidation because a mutation on a pleiotropic gene may have an effect on some, all, or none of its traits. Therefore, mutations in a single gene may cause multiple syndromes or only cause disease in some of the biological processes the gene mediates. Establishing which genotypes are responsible for the perturbed phenotype of interest is not straightforward.

*Genes can influence one another* in several ways; genes can interact synergistically, (as in *epistasis*), or they can modify one another (e.g. the expression of one gene might affect the expression of another). Cystic fibrosis and Becker muscular dystrophy, previously considered classical examples of Mendelian patterns of inheritance, are now believed to be caused by a mutation of one gene which is modified by other genes [57], [58]. Thus, even simple Mendelian diseases can lead to complex genotype-phenotype associations [59].

*Environmental factors* (e.g. diet, infection by bacteria) are also major determinants of disease phenotype expression often acting in combination with other genotype-phenotype association confounders (i.e. pleiotropy and gene modifiers). In fact, most common diseases such as cancer, metabolic, psychiatric and cardio-vascular disorders (e.g. diabetes, schizophrenia and hypertension) are believed to be caused by several genes (multigenic) and are affected by several environmental factors [60].

### 4.2 Studying the Molecular Basis of Disease

Much can be learned from documenting the genes associated with a particular disease (e.g. identifying risk factors that might be used for diagnostic purposes). Yet, to understand the biological details of pathogenesis and disease progression and to subsequently develop methods for prevention, treatment and even diagnosis, it is necessary to identify the molecules and the mechanisms triggering, participating, and controlling the perturbed biological process. Deciphering the molecular mechanisms leading to diseased states is an even bigger challenge than elucidating the genetic basis of complex diseases [61]. Even when the genetic basis of a disease is well understood, not much is known about the molecular details leading to the disorders.

#### 4.2.1 The role of protein interactions in disease

Protein interactions provide a vast source of molecular information; their interactions (with one another, DNA, RNA, or small molecules) are involved in metabolic, signaling, immune, and gene-regulatory networks. Since protein interactions mediate the healthy states in all biological processes, it follows that they should be the key targets of the molecular-based studies of biological diseased states. Disease-causing mutations affecting protein interactions can lead to disruptions in protein-DNA interactions, protein misfolds, new undesired interactions, or can enable pathogen-host protein interactions.

*Protein-DNA interaction disruptions* are most clearly illustrated by the p53 tumor suppressor protein and its role in cancer. Mutations on p53's DNA-binding domain destroy its ability to bind to its target DNA sequences, thus preventing transcriptional activation of several anti-cancer mechanisms it mediates (e.g. apoptosis, genetic stability, and inhibition of angiogenesis).

*Protein misfolding* can result in disruptions of protein-protein interactions, as occurs in the Von Hippel-Lindau syndrome (VHL)—VHL is a rare condition in which hemangioblastomas are formed in the cerebellum, spinal cord, kidney, and retina. A mutation from Tyrosine to Histidine at residue 98 on the binding site disrupts binding of the VHL protein to the hypoxia-inducible factor (HIF) protein. As a result, the VHL protein no longer degrades the HIF protein, which leads to the expression of angiogenic growth factors and local proliferation of blood vessels [62], [63].

*New undesired protein interactions* are the main causes of several diseases, including Huntington's disease (see introduction), cystic fibrosis, and Alzheimer's disease. New interactions alter homeostasis since they can lead to the loss of vital cellular functions (due to misfolding and aggregation) and can cause cytotoxicity [11].

*Pathogen-host protein interactions* also play a key role in bacterial and viral infections by facilitating the hijacking of the host's metabolism for microbial need. The interaction between the Human papillomavirus (HPV) and its host provides one of the most striking examples of the centrality of protein interactions in infectious diseases. HPV infection occurs in a large fraction of the population (75–80% of Americans [64]) by generating lesions of the anogenital tract and for some it leads to cancer. Upon infection, the HPV genome is frequently integrated into the host genome, but only two viral genes (*E6* and *E7*) are retained and expressed. Remarkably, the interactions of only two viral proteins with the host's proteins are enough to cause HPV-induced carcinogenesis. E6 and E7 bypass the immune system by interacting with important negative cell regulatory proteins to target them for degradation and thus, inactivation. These two proteins also inhibit cellular terminal differentiation, induce cellular transformation and immortalization of the host cells, and direct the proliferation of the tumorigenically-transformed cells [65].

#### 4.2.2 Using PPI networks to understand disease

*PPI networks can help identify novel pathways* to gain basic knowledge of disease. Note that pathways are different from PPI networks. PPI networks map the physical or functional interaction between protein pairs resulting in a complex grid of connections (Figure 1). Pathways, on the other hand, represent genetic, metabolic, signaling, or neural processes as a series of sequential biochemical reactions where substrates are changed in a linear fashion. For instance, the glycolysis pathway maps the conversion of glucose to pyruvate through a linear chain of ten different steps.

Pathway analysis alone cannot uncover the molecular basis of disease. When performing pathway analysis to study disease, differential expression experiments are the main source of protein candidates. However, most of the gene expression candidates are useless to pathway-based analysis of disease because the majority of human genes have not been assigned to a pathway. Protein interaction networks can be used to identify novel pathways. Protein interaction subnetworks tend to group together the proteins that interact in functional complexes and pathways [66]. Thus, new methods are being developed to accurately extract interaction subnetworks to yield pathway hypotheses that can be used to understand different aspects of disease progression [67], [68]. See Table 1 for useful resources incorporating pathway and PPI information in disease elucidation.

**Table 1:** Pathway databases with disease information.

Resource	Featured organisms	Disease information	Website
KEGG	Yeast, mouse, human	Comprehensive	http://www.genome.jp/kegg/disease/
REACTOME	Human+20 other species	Sparse	http://reactome.org/
SMPDB	Human	Small molecules' Metabolic disease pathways	http://www.smpdb.ca
PharmGKB	Human	Gene-drug-disease relationships	http://www.pharmgkb.org/index.jsp
NetPath	Human	10 immune and 10 cancer pathways	http://www.netpath.org/index.html

Mapping interactomes provide the opportunity to identify disease pathways by identifying key subnetworks. In 2005, Rual *et al.* [69] mapped the human protein interactome. Below are some of the findings that have been uncovered when combining PPI and pathway analysis since then.

- Over 39,000 protein interactions have been identified in the human cell [70].
- Disease genes are generally non-essential and occupy peripheral positions in the human interactome [71], although, in a few diseases like cancer, disease genes tend to encode highly-connected proteins (hubs) [72], [73].
- Disease genes tend to cluster together and co-occur in central network locations [6].
- Proteins involved in similar phenotypes (e.g. all cancer proteins) are highly interconnected [73].
- Viral networks differ significantly from cellular networks, which raises the hypothesis that other intracellular pathogens might also have distinguishing topologies [74].
- Etiologically unrelated diseases often present similar symptoms because separate biological processes often use common molecular pathways [75].

*PPI networks can be used to explore the differences* between healthy and diseased states. Building interaction networks for systems under different conditions (e.g. wild type vs. mutant, presence of environmental factor vs. its absence) might be the key to understanding the differences between healthy and pathological states. The work by Charlesworth *et al.* [76] on the perturbation of the canonical pathways and networks of interactions when humans are exposed to cigarette smoke illustrates the potential of such approaches. As one might expect, this study found that the smoking-susceptible genes were overrepresented in pathways involved in several aspects of cell death (cell cytotoxicity, cell lysis), cancer (e.g. tumorigenesis), and respiratory functions. A somewhat more unexpected finding, however, confirmed that exposure to the smoke environmental factor affected a large subnetwork of proteins involved in the immune-inflammatory response. This study gave new insights into how smoke causes disease: the exogenous toxicants in smoke perturb several protein interactions in the healthy cell state, thereby depressing the immune system, while disrupting the inflammation response. The study also explained why smoking cessation has some immediate health benefits; eliminating smoke exposure reverses the alterations at the transcriptomic level and restores the majority of normal protein interactions.

*Protein interaction studies play a major role in the prediction of genotype-phenotype* associations while also identifying new disease genes. The identification of disease-associated interacting proteins also identifies potentially interesting disease-associated gene candidates (i.e. the genes coding for the interacting proteins are putative disease-causing genes). One of the best ways to identify novel disease genes is to study the interaction partners of known disease-associated proteins [77]. Gandhi *et al.* [78] found that mutations on the genes of interacting proteins lead to similar disease phenotypes, presumably because of their functional relationship. Therefore, protein interactions can be used to prioritize gene candidates in studies investigating the genetic basis of disease [79]. Others have used the properties of protein interaction networks to differentiate disease from non-disease proteins. Based on this approach, Xu *et al.* [80] devised a classifier based on several topological features of the human interactome to predict genes related to disease. The classifier was trained on a set of non-disease and a set of disease genes (from OMIM) and applied to a collection of over 5,000 human genes. As a result, 970 disease genes were identified, a fraction of which were experimentally validated.

*New diagnostic tools can result from genotype-phenotype associations* established through PPIs. The genes of interacting proteins can be studied to identify the mutation(s) leading to the interaction disruptions seen in healthy individuals or to the creation of new interactions only present in the diseased states. For example, Rossin *et al.* used genome-wide association studies (GWAS) to identify regions with variations that predispose immune-mediated diseases [81]. The GWAS studies provided a list of proteins found to interact in a preferential manner. The resulting disease single-nucleotide polymorphisms identified by GWAS studies such as that by Rossin *et al.* can be eventually incorporated into genotyping diagnostic tools.

*Identifying disease subnetworks*, *and in turn pathways that get activated in diseased states*, *can provide markers* to create new prognostic tools. For instance, using a protein-network-based approach, Chuang *et al*. [66] identified a set of subnetwork markers that accurately classify metastatic vs. non-metastatic tumors in individual patients. Metastasis is the leading cause of death in patients with breast cancer. However, a patient's risk for metastasis cannot be accurately predicted and it is currently only estimated based on other risk factors. When metastasis is deemed likely, breast cancer patients are prescribed aggressive chemotherapy, even when it might be unnecessary. By integrating protein networks with cancer expression profiles, the authors identified relevant pathways that become activated during tumor progression, which discriminate metastasis better than markers previously suggested by studies using differential gene expression alone.

*Disease networks can inform drug design* by helping suggesting key nodes as potential drug targets. Drug target identification constitutes a good example of the potential of integrating structural data with high-throughput data [82]. The structural details on binding or allosteric sites can be used to design molecules to affect protein function. On the other hand, reconstruction of the different protein networks (signaling, metabolic, regulatory, etc.) in which the potential target is involved can help predict the overall impact of the disruption. If, for example, the target is a hub (a highly connected protein), its inhibition may affect many activities that are essential for the proper function of the cell and might thus be unsuitable as a drug target. On the other hand, less connected nodes (e.g. nodes affecting a single disease pathway) could constitute vulnerable points of the disease-related network, which are better candidates for drug targets. The work by Yildirim and Goh [83] illustrates the advantages of evaluating drugs within the context of cellular and disease networks. This group created a drug-target network to map the relationships between the protein targets of all drugs and all disease-gene products. The topological analysis of the human drug-target network revealed that (i) most drugs target currently known targets; (ii) only a small fraction of disease genes encodes drug-target proteins; (iii) current drugs do not target diseases equally but only address some regions of the human disease network; and (iv) most drugs are palliative—they treat the symptoms not the cause of the disease, which largely reflects our lack of knowledge regarding the molecular basis of diseases such that for many pathologies we can only treat the symptoms but not cure them.

## 5. Summary—Trends in the Translational Characterization of Human Disease

We are still quite far from understanding the etiology of most diseases. Further advances on relevant experimental technology (e.g. genetic linkage, protein interaction prediction), along with integrative computational tools to organize, visualize, and test hypotheses should provide a step forward in that direction. More than ten years after the completion of the human genome project, it is clear that our approach to human disease elucidation needs to change. The $3-billion human “book of life” and the $138-million effort to catalog the common gene variants relevant to disease have so far failed to deliver the wealth of biological knowledge of human diseases and the subsequent personalization of medicine the scientific community expected [84].

To date, biomedical research of the etiology of disease has largely focused on identifying disease-associated genes. But, the molecular mechanisms of pathogenesis are extremely complex; gene-products interact in different pathways and multiple genes and environmental factors can affect their expression and activity. Likewise, the same proteins may participate in different pathways and mutations on their genes may or may not affect some or all of the biological processes they mediate. Thus, gene-disease associations cannot be straightforwardly deduced and their usefulness alone (in the absence of a molecular context) in elucidating the biology of healthy phenotype disruptions is questionable. Evidence is accumulating to suggest that in the majority of cases illnesses are traceable to a large number of genes affecting a network or pathway. The effects on healthy phenotype disruption may vary from one individual to another based on the person's gene variants and on how disruptive the alterations might be to the network [85].

To achieve a comprehensive genotype-phenotype understanding of disease, translational research should be conducted within a framework integrating methodologies for uncovering the genetics with those investigating the molecular mechanisms of pathogenesis. In fact, the studies yielding the most biological insight into disease to which we alluded in this chapter were those which implemented a combined genotype-phenotype approach; those studies identified the disease-susceptible genes and investigated their network of interactions and affected pathways. As a result, the combined approaches managed to explain known clinical observations while also suggesting new mechanisms of pathology.

PPI analysis provides an effective means to investigate biological processes at the molecular level. Yet, any conclusions obtained based on PPI methods must be validated since these methods are subject to limitations inherent to the nature of data collection and availability. First, one must be aware that the roles of protein interactions are context-specific (tissue, disease stage, and response). Thus, two proteins observed to interact *in vitro* might not interact *in vivo* if they are localized in different cell compartments. Even when in common cell compartments, protein abundance or presence of additional interactors might affect whether the interaction occurs at all. Second, most of the PPI methodologies use a simplistic ‘static’ view of proteins and their networks. In reality, proteins are continuously being synthesized and degraded. The kinetics of processes and network dynamics need to be considered to achieve a complete understanding of how the disruptions of protein interactions lead to disease. Third, human PPIs are often predicted based on homology and from studies investigating disease in other organisms. The same mechanisms of interaction might or might not exist in the organism of interest or their regulation and phenotypic effects might be different. Ideally, since network and structural approaches are complementary, the combination of network studies with a more detailed structural analysis has the potential to enhance the study of disease mechanisms and rational drug design.

Currently, in the PPI field, a large number of studies focus on the topological characterization of organisms' interactomes. Those studies have yielded valuable information regarding general trends of molecular organization and their differences across genomes. To gain a deeper understanding of individual diseases, however, the trend needs to move from global characterizations to disease-specific interactomes. Phenotype-specific interaction network analyses should help identify subnetworks mapping to pathways that can be targeted therapeutically and point to key molecules essential to the biological function under study. Since disease inferences are as good as the modeled PPI networks, the ontologies used by PPI resources need to be expanded to better describe disease phenotypes, cytological changes, and molecular mechanisms.

## 6. Exercises

**Objective: To investigate Epstein-Barr Virus (EBV) pathogenesis using protein-protein interactions**

EBV is a member of the herpesvirus family and one of the most common human viruses. According to the CDC, in the United States around 95% of adults have been infected by EBV. Upon infection in adults, EBV replicates in epithelial cells and establishes latency in B lymphocytes, eventually causing infectious mononucleosis 35%–50% of the time and sometimes cancer [86]. In the next four sections, your goal will be to study the interactions among EBV proteins and between the virus and its host (using the EBV-EBV and EBV-human interactomes respectively) as a means to investigate how EBV leads to disease at the molecular level.

*Datasets:*

The following datasets were adapted with permission from [87]

- Dataset S1: EBV interactome
- Dataset S2: EBV-Human interactome

*Software requirements:*

Download and install Cytoscape (http://www.cytoscape.org, [88]) locally.

*Note:*

The instructions below correspond to Cytoscape v. 2.8.0; but, should be applicable to future releases.

**I. Visualize the EBV interactome using Cytoscape**

- **Import Dataset S1 into cytoscape**
  - *Select File ->Import ->Network (Multiple File Types)*
  - *Click the “Select” button* to browse to Dataset S1's location
  - *Click “Import”*
- **Change the network layout**
  - *Click on View->Hide data panel*
  - *Click the 1∶1 magnifying glass icon* to zoom out to display all elements of the current network”
  - *Select Layout->Cytoscape Layouts->Force-directed (unweighted) Layout*
- **Format the nodes and edges**
  - *Select View->Open Vizmapper*
  - *Choose the “Default” Current Visual Style*
  - *Click on the pair of connected nodes icon in the “Defaults” box*
  - *Scroll down on the resulting dialog* to change the following default visual properties:
    NODE__SIZE = 20
    NODE_FONT_SIZE = 20
    NODE_LABEL_POSITION = (Node Anchor Points) SOUTH
  - *Note*: Feel free to click and drag any nodes with labels that overlap to increase visual clarity.
- **Print the EBV interactome**
  - Select File->Export->Current Network View as Graphics

*Answer the following questions:*

- How many nodes and edges are featured in this network?
- How many self interactions does the network have?
- How many pairs are not connected to the largest connected component?
- Define the following topological parameters and explain how they might be used to characterize a protein-protein interaction network: node degree (or average number of neighbors), network heterogeneity, average clustering coefficient distribution, network centrality.

**II. Characterize the EBV-Human interactome**

Import Dataset S2 into cytoscape to create a map of the EBV-Human interactome. Format and output the network according to steps A through D in part I.

*Answer the following questions:*

- How many unique proteins were found to interact in each organism?
- How many interactions are mapped?
- How many human proteins are targeted by multiple (i.e. how many individual human proteins interact with >1) EBV proteins?
- How does identifying the multi-targeted human proteins help you understand the pathogenicity of the virus? —Hint: Speculate about the role of the multi-targeted human proteins in the virus life cycle.
- How might you test the predictions you formulated above?

**III. Characterize the topological properties of the human proteins that are targeted by EBV**

 Use the topological information provided for you in Table 2 to investigate whether the EBV-targeted Human Proteins (ET-HPs) differ from the average human protein.

**Table 2:** Topological properties of human proteins for exercise III.

Average topological property	ET-HP	Random human protein
Degree	15±2	5.9±0.1
Number of components	4	12.6±0.25
Nodes in largest component	1,112	521±5
Distance to other proteins	3.2±0.1	4.03±0.01

*Answer the following questions:*

- Based on the ‘degree’ property, what can you deduce about the connectedness of ET-HPs? What does this tell you about the kind of proteins (i.e. what type of network component) EBV targets?
- What do the number and size of the largest components tell you about the inter-connectedness of the ET-HP subnetwork?
- Why is distance relevant to network centrality? What is unusual about the distance of ET-HPs to other proteins and what can you deduce about the importance of these proteins in the Human-Human interactome?
- Based on your conclusions from questions i–iii, explain why EBV targets the ET-HP set over the other human proteins and speculate on the advantages to virus survival the protein set might confer.

**IV. Integrating knowledge from three different interactomes**

*Answer the following questions:*

- The Rta protein is a transactivator that is central to viral replication in EBV. When Rta is co-expressed with the LF2 protein replication attenuates and the virus establishes latency. Solely based on the EBV-EBV network, formulate a hypothesis to explain how LF2 may be driving EBV to latency suggesting at least one molecular mechanism by which LF2 may inactivate Rta.
- Why is establishing latency (opposed to promoting rapid replication of viral particles) an effective mechanism of virus infection?
- Assign putative functions to EBV's SM and EBNA3A proteins based on the function of the human proteins with which they interact—Hint: Locate these proteins in the EBV-Human network. What clinical observation (see the introductory paragraph to section 6. Exercises) might these proteins' subnetworks explain?

Answers to the Exercises can be found in Text S1.

**Further Reading**

- Chen JY, Youn E, Mooney SD (2009) Connecting protein interaction data, mutations, and disease using bioinformatics. Methods Mol Biol 541: 449–461.
- Nussinov R, Schreiber G (2009) Computational protein-protein interactions. Boca Raton: CRC Press.
- Ideker T, Sharan R (2008) Protein networks in disease. Genome Res 18: 644–652.
- Juan D, Pazos F, Valencia A (2008) Co-evolution and co-adaptation in protein networks. FEBS Lett 582: 1225–1230.
- Panchenko A, Przytycka T (2008) Protein-protein interactions and networks: identification, computer analysis, and prediction. London: Springer.
- Klussmann E, Scott J, Aandahl EM (2008) Protein-protein interactions as new drug targets. Berlin: Springer.
- Kann MG (2007) Protein interactions and disease: computational approaches to uncover the etiology of diseases. Brief Bioinform 8: 333–346.
- Shoemaker BA, Panchenko AR (2007) Deciphering protein-protein interactions. Part II. Computational methods to predict protein and domain interaction partners. PLoS Comput Biol 3: e43. doi:10.1371/journal.pcbi.0030043

**Glossary**

**Mendelian traits or diseases**, named after Gregor Mendel, are the traits inherited and controlled by a single gene.

**Positional cloning** is a method to find the gene producing a specific phenotype in an area of interest in the genome. The first step of positional cloning is linkage analysis, in which the gene is mapped using a group of DNA polymorphisms from families segregating the disease phenotype.

**Epistasis** refers to the phenomenon in which one gene masks the phenotypic effect of another.

**Angiogenesis** is the physiological process leading to growth of new blood vessels. Angiogenesis is a normal and vital process in growth, development, and wound healing; but it is also a fundamental step in the transition of tumors from a dormant to a malignant state.

**Hemangioblastomas** are tumors of the central nervous system that originate from the vascular system.

## Supporting Information

Text S1 Answers to Exercises.
(DOCX)

Dataset S1 EBV Interactome Data.
(SIF)

Dataset S2 EBV-Human Interactome Data.
(SIF)

Figure S1 EBV Interactome Map.
(PDF)

Figure S2 EBV-Human Interactome Map.
(PDF)
