# Supplementary material for: Chapter 4: Protein Interactions and Disease
Source: PLoS Comput Biol. 2012 Dec 27;8(12):e1002819. doi: 10.1371/journal.pcbi.1002819 (PMC3531279; doi:10.1371/journal.pcbi.1002819)
Supplement: Text S1 — Answers to Exercises. (DOCX) [file pcbi.1002819.s001.docx]

**Protein Interactions and Disease**

**Exercises** **(With Answers)**

Mileidy W. Gonzalez and Maricel G. Kann

**Objective: To investigate Epstein-Barr Virus (EBV) pathogenesis using protein-protein interactions**

EBV is a member of the herpesvirus family and one of the most common human viruses. According to the CDC, in the United States around 95% of adults have been infected by EBV. Upon infection in adults, EBV replicates in epithelial cells and establishes latency in B lymphocytes, eventually causing infectious mononucleosis 35%-50% of the time and sometimes cancer [[1](#_ENREF_1)]. In the next four sections, your goal will be to study the interactions among EBV proteins and between the virus and its host (using the EBV-EBV and EBV-human interactomes respectively) as a means to investigate how EBV leads to disease at the molecular level.

Datasets:

The following datasets were adapted with permission from [[2](#_ENREF_2)]

- Dataset S1: EBV interactome
- Dataset S2: EBV-Human interactome

Software requirements:

Download and install Cytoscape (<http://www.cytoscape.org>, [[3](#_ENREF_3)]) locally.

Note:

The instructions below correspond to Cytoscape v. 2.8.0; but, should be applicable to future releases.

1. **Visualize the EBV interactome using Cytoscape**

**A. Import Dataset S1 into cytoscape**

*Select File ->Import ->Network (Multiple File Types)*

*Click the “Select” button* to browse to Dataset 1’s location

*Click “Import”*

**B. Change the network layout**

*Click on View-> Hide data panel*

*Click the 1:1 magnifying glass icon* to zoom out to display all elements of the current network”

*Select Layout-> Cytoscape Layouts-> Force-directed (unweighted) Layout*

**C. Format the nodes and edges**

*Select View-> Open Vizmapper*

*Choose the “Default” Current Visual Style*

*Click on the pair of connected nodes icon in the “Defaults” box*

*Scroll down on the resulting dialog* to change the following default visual properties:

NODE__SIZE = 20

NODE_FONT_SIZE = 20

NODE_LABEL_POSITION = (Node Anchor Points) SOUTH

Note: Feel free to click and drag any nodes with labels that overlap to increase visual clarity.

**D. Print the EBV interactome**

Select File-> Export->Current Network View as Graphics

View Figure S1.

Answer the following questions:

1. How many nodes and edges are featured in this network?

52 nodes 62 edges.

1. How many self interactions does the network have?

11.

1. How many pairs are not connected to the largest connected component?

12.

1. Define the following topological parameters and explain how they might be used to characterize a protein-protein interaction network: node degree (or average number of neighbors), network heterogeneity, average clustering coefficient distribution, network centrality.

A **node’s degree** represents the number of direct interactions between a given node and its neighbors. The average number of neighbors indicates the average connectivity of a network.

The **network heterogeneity** reflects the tendency of a network to contain hub nodes. Identifying hubs is an important step in the characterization of a network because they often mediate central roles in pathways.

The **clustering** **coefficient** is a ratio N/M, where N is the number of edges between the neighbors of n, and M is the maximum number of edges that could possibly exist between the neighbors of n. The average clustering coefficient distribution gives the average of the clustering coefficients for all nodes n with k neighbors for k = 2,…. Network clustering coefficients are useful in identifying functional modules and in measuring their effect on network organization.

The **average shortest path length**, also known as the characteristic path length, gives the expected distance between two connected nodes.

**Network centrality** refers to the relative importance of a node in a network.

1. **Characterize the EBV-Human interactome**

Import Dataset S2 into cytoscape to create a map of the EBV-Human interactome. Format and output the network according to steps A through D in part I.

View Figure S2.

Answer the following questions:

- - 1. How many unique proteins were found to interact in each organism?

40 EBV, 112 human.

- - 1. How many interactions are mapped?

173 interactions.

- - 1. How many human proteins are targeted by multiple (i.e. how many individual human proteins interact with >1) EBV proteins?

There are 24 multi-targeted human proteins in the EBV-human interactome.

- - 1. How does identifying the multi-targeted human proteins help you understand the pathogenicity of the virus? —Hint: Speculate about the role of the multi-targeted human proteins in the virus life cycle.

The fact that multiple EBV proteins target single human proteins suggests that those proteins and their interactions are crucial to the virus life cycle. This is useful because it gives us a way to target key proteins for further study.

- - 1. How might you test the predictions you formulated above?

To test the validity of the above predictions one may conduct experimental (e.g. gene knockout and/or structural) studies to test whether disruption of individual interactions reduces virulence.

**III. Characterize the topological properties of the human proteins that are targeted by EBV**

Use the topological information provided for you in the table below to investigate whether the EBV-targeted Human Proteins (ET-HPs) differ from the average human protein.

| **Average topological property** | **ET-HP** | **Random human protein** |
| --- | --- | --- |
| Degree | $15\pm2$ | $5.9\pm0.1$ |
| Number of components | $4$ | $12.6\pm0.25$ |
| Nodes in largest component | $1,112$ | $521\pm5$ |
| Distance to other proteins | $3.2\pm0.1$ | $4.03\pm0.01$ |

Answer the following questions:

1. Based on the ‘degree’ property, what can you deduce about the connectedness of ET-HPs? What does this tell you about the kind of proteins (i.e. what type of network component) EBV targets?

The average degree of ET-HPs is significantly higher than that of a random human protein, which suggests that ET-HPs are highly connected proteins: i.e. hubs.

1. What do the number and size of the largest components tell you about the inter-connectedness of the ET-HP subnetwork?

The ET-HP subnetwork has fewer components with more nodes than the average random network, which tells us that the ET-HP subnetwork is highly interconnected.

1. Why is distance relevant to network centrality? What is unusual about the distance of ET-HPs to other proteins and what can you deduce about the importance of these proteins in the Human-Human interactome?

Distance allows us to measure network centrality. Since the average distance of ET-HPs is smaller than that of average proteins we can deduce that ET-HPs are important components of the entire human interactome.

1. Based on your conclusions from questions i-iii, explain why EBV targets the ET-HP set over the other human proteins and speculate on the advantages to virus survival the protein set might confer.

EBV targets the proteins in the ET-HP set because they are highly connected proteins that are central to the host’s metabolism. Furthermore, by targeting this set, the virus has chosen proteins that form highly interconnected components that in all likelihood maximize the efficacy of relevant biological processes. The choice of proteins reflects the set of proteins that best allow the virus to hijack the host metabolism to use it to its own purposes.

**IV. Integrating knowledge from three different interactomes**

Answer the following questions:

1. The Rta protein is a transactivator that is central to viral replication in EBV. When Rta is co-expressed with the LF2 protein replication attenuates and the virus establishes latency. Solely based on the EBV-EBV network, formulate a hypothesis to explain how LF2 may be driving EBV to latency suggesting at least one molecular mechanism by which LF2 may inactivate Rta.

The EBV-EBV network shows LF2 directly interacting with Rta.

Hypothesis: when Lta interacts with Rta it inhibits its activity such that the virus no longer replicates, but becomes latent.

Mechanisms by which latency may be triggered: i) Lta prevents the activation of Rta by another molecule(s) by competitive binding or through conformational changes in Rta; ii) Lta may decrease Rta levels by downregulating its expression; iii) Lta may inhibit Rta promoter activation; iv) Lta may change Rta’s subcellular localization.

ii. Why is establishing latency (opposed to promoting rapid replication of viral particles) an effective mechanism of virus infection?

Establishing latency upon infection has the following advantages.

- It allows the virus to remain undetected (below the radar of) the immune system).
- It keeps the viral genome from being digested by cellular ribozymes.
- It facilitates virus persistence, allowing the virus to stay within the host for years or indefinitely.
- The virus can reactivate quickly upon favorable conditions (e.g. external signal, depressed immune system), thus producing large amounts of viral progeny without the host being infected by new outside viral particles.

1. Assign putative functions to EBV’s SM and EBNA3A proteins based on the function of the human proteins with which they interact—Hint: Locate these proteins in the EBV-Human network. What clinical observation (see the introductory paragraph to the Exercises) might these proteins’ subnetworks explain?

Three out of four of SM’s and both of EBNA3A’s interacting partners mediate biological processes involved in cancer. Thus, likely, SM and EBNA3A play a functional role in cancer, which explains the observation that in some cases EBV can also lead to cancer in its infected hosts.

**References**

1. CDC (2006) Epstein-Barr Virus and Infectious Mononucleosis. Center for Disease Control and Prevention/National Center for Infectious Diseases.

2. Calderwood MA, Venkatesan K, Xing L, Chase MR, Vazquez A, et al. (2007) Epstein-Barr virus and virus human protein interaction maps. Proc Natl Acad Sci U S A 104: 7606-7611.

3. Smoot ME, Ono K, Ruscheinski J, Wang PL, Ideker T (2011) Cytoscape 2.8: new features for data integration and network visualization. Bioinformatics 27: 431-432.
