# Supplementary figures and images for: Chapter 4: Protein Interactions and Disease
Source: PLoS Comput Biol. 2012 Dec 27;8(12):e1002819. doi: 10.1371/journal.pcbi.1002819 (PMC3531279; doi:10.1371/journal.pcbi.1002819)

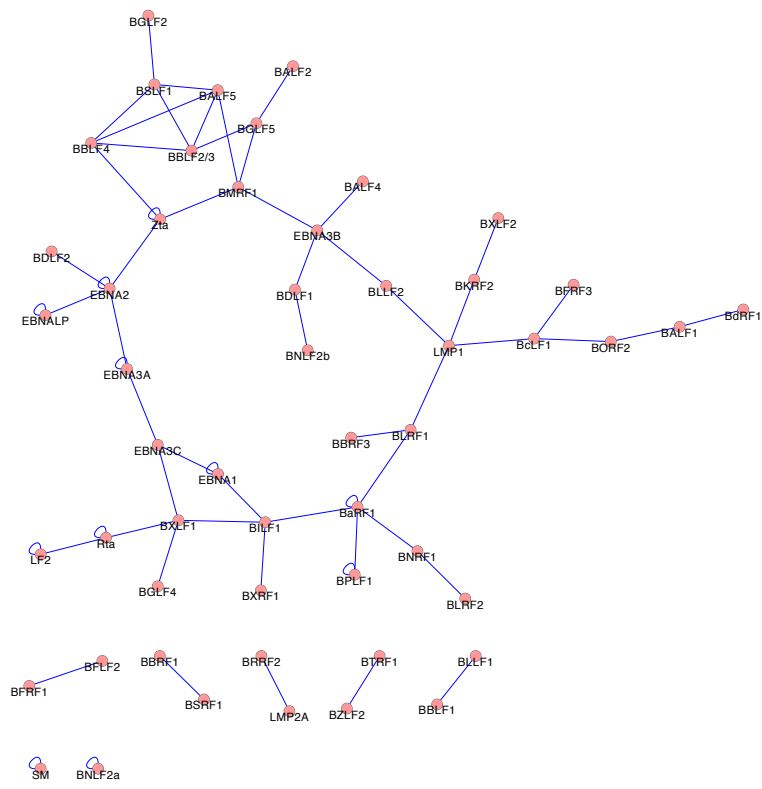

Supplement: Figure S1 — EBV Interactome Map. (PDF) [file pcbi.1002819.s004.pdf]

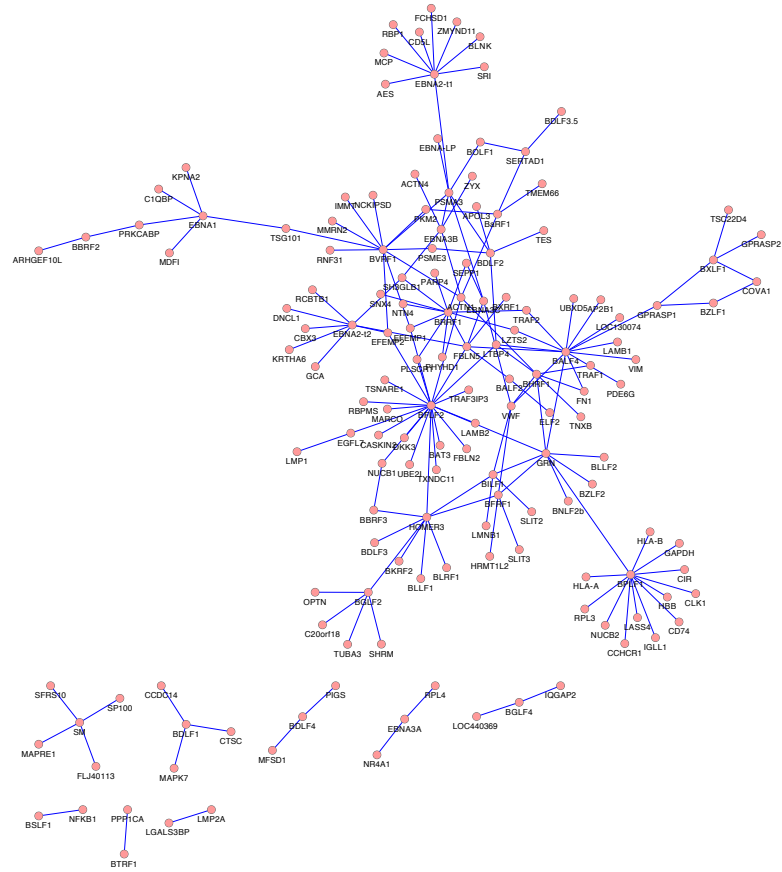

Supplement: Figure S2 — EBV-Human Interactome Map. (PDF) [file pcbi.1002819.s005.pdf]
